# Superantigens Modulate Bacterial Density during *Staphylococcus aureus* Nasal Colonization

**DOI:** 10.3390/toxins7051821

**Published:** 2015-05-22

**Authors:** Stacey X. Xu, Katherine J. Kasper, Joseph J. Zeppa, John K. McCormick

**Affiliations:** 1 Department of Microbiology and Immunology, Schulich School of Medicine and Dentistry, Western University, London, ON N6A 5C1, Canada; E-Mails: sxu29@uwo.ca (S.X.X.); kkasper@uwo.ca (K.J.K.); jzeppa3@uwo.ca (J.J.Z.); 2 Lawson Health Research Institute, London, ON N6A 5C1, Canada

**Keywords:** superantigen, *Staphylococcus aureus*, nasal colonization, humanized mice

## Abstract

Superantigens (SAgs) are potent microbial toxins that function to activate large numbers of T cells in a T cell receptor (TCR) Vβ-specific manner, resulting in excessive immune system activation. *Staphylococcus aureus* possesses a large repertoire of distinct SAgs, and in the context of host-pathogen interactions, staphylococcal SAg research has focused primarily on the role of these toxins in severe and invasive diseases. However, the contribution of SAgs to colonization by *S. aureus* remains unclear. We developed a two-week nasal colonization model using SAg-sensitive transgenic mice expressing HLA-DR4, and evaluated the role of SAgs using two well-studied stains of *S. aureus*. *S. aureus* Newman produces relatively low levels of staphylococcal enterotoxin A (SEA), and although we did not detect significant TCR-Vβ specific changes during wild-type *S. aureus* Newman colonization, *S. aureus* Newman Δ*sea* established transiently higher bacterial loads in the nose. *S. aureus* COL produces relatively high levels of staphylococcal enterotoxin B (SEB), and colonization with wild-type *S. aureus* COL resulted in clear Vβ8-specific T cell skewing responses. *S. aureus* COL Δ*seb* established consistently higher bacterial loads in the nose. These data suggest that staphylococcal SAgs may be involved in regulating bacterial densities during nasal colonization.

## 1. Introduction

*Staphylococcus aureus* is recognized as a major human pathogen causing a range of illnesses from superficial skin infections to invasive diseases including bacteremia, sepsis, pneumonia, and endocarditis [1]. Within the healthcare setting, *S. aureus* infections are particularly serious, including infection by methicillin-resistant *S. aureus* (MRSA) strains, and this pathogen is now the most significant cause of serious infections in the United States [1,2,3,4].

Despite the massive burden of disease, asymptomatic carriage by *S. aureus* is pervasive within human populations, being found most typically on the skin and in the nasal cavity. Nasal carriers have been defined as persistent, intermittent, or non-carriers, and although the definitions for each group can vary between studies, ~50% of the population are persistent or intermittent carriers, with some studies showing even higher levels of colonization [4,5]. *S. aureus* typically resides within the vestibulum nasi of the anterior nares and has been found colonizing the cornified layer of stratified squamous epithelium, keratinized surfaces and mucous debris, as well as hair follicles of the human nose [6]. Given these anatomical findings, it is not surprising that *S. aureus* is able to bind to both keratinized cells and desquamated nasal epithelia. These act as key host cells upon which *S. aureus* initiates colonization [7,8]. Nasal carriers of *S. aureus* are generally asymptomatic and healthy, forming a commensal relationship with the bacteria. However, colonization status increases the risk of a severe infection from the carrier strain, although nasal carriers tend to have a better prognosis in the event of a staphylococcal infection [9]. This is thought to be due to specific immunity built up against the colonizing strain [10]. Bacterial components contributing to staphylococcal colonization are multifactorial and include host genetic factors that influence carrier status [11], as well as a variety of bacterial adhesins and cell-wall associated factors such as clumping factor B (ClfB) [12], wall teichoic acids [13], surface protein SasG [14], and iron-regulated surface determinant A (IsdA) [15].

Superantigens (SAgs) are a group of toxins produced by bacteria including *S. aureus* that mediate interactions between peptide-MHC class II and the CDR2 loop of the variable chain of the T cell receptor [16]. As SAg-mediated T cell activation is not dependent on the antigenic peptide presented in the MHC class II molecule, this response can activate very large numbers of the exposed T cell population and may, in rare cases, lead to a ‘cytokine storm’ disease known as the toxic shock syndrome (TSS). These toxins have also been implicated in many other diseases including infectious endocarditis, Kawasaki disease, atopic dermatitis, and various autoimmune diseases [16,17]. To date, more than twenty *S. aureus* SAgs have been identified including an operon of SAgs known as the enterotoxin gene cluster (*egc*) encoding staphylococcal enterotoxins (SE) G, I and SE-like (SEl) M, N, O and U [16,18].

Assessment of the humoral response from *S. aureus* colonized individuals have shown that persistent carriers produce high titer neutralizing antibodies with specificity for the SAgs produced by the carrier strain [10,19]. Additionally, assessment of antibody titers to seven different staphylococcal SAgs showed increased antibodies to toxic shock syndrome toxin-1 (TSST-1) and staphylococcal enterotoxin A (SEA) in persistent carriers compared with non-carriers [20]. Epidemiological studies of clinical isolates revealed a high prevalence of *egc* SAgs [21], as well as a negative correlation of these toxins with severe septic shock [22]. Nasal swabs from persistent carriers revealed that *sea*, *sec* and *sel-o* were actively transcribed; however, neutralizing antibodies against SEA and SEC, but not SEl-O, were detected in this cohort [23]. It was concluded that the robust antibody response against the non-*egc* SAgs was due to minor infections rather than colonization [23]. Also, vaccination of mice with SAg toxoids seems to protect only against the early phase of colonization (days 1 and 3) [24] suggesting that SAgs may be involved in initial colonization, but further implications are difficult to extrapolate. Collectively, these studies have shed light on the highly complex nature of nasal colonization and hinted at a role for SAgs in nasal carriage in humans, and mouse infection models. However, the role of SAgs during nasal colonization, either for establishing initial colonization, or involvement in dissemination, has not been experimentally addressed.

Human studies reveal low levels of bacteria in the nose, with 10^1^–10^4^ colony forming units (CFU) of *S. aureus* being typically isolated from nasal swabs [25]. We hypothesized that secreted SAgs may act as ‘checkpoints’ of colonization in order to maintain this state of commensalism and to prevent high bacterial densities through activation of the immune system, and subsequent elimination of invasive organisms. In order to test this hypothesis, we created isogenic SAg deletions of two well-characterized strains of *S. aureus*, and tested these strains against their wild-type counterparts in a SAg-sensitized murine model of staphylococcal nasal colonization.

## 2. Results

### 2.1. SAg Deletion Strains Have Reduced Superantigen Production and Activity in vitro

**Figure 1:**
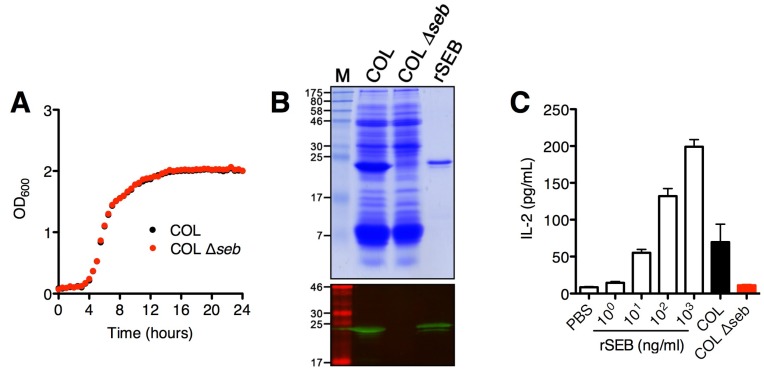
*S. aureus* COL Δ*seb* does not produce staphylococcal enterotoxin B (SEB) and has greatly reduced superantigenic activity. (**A**) Growth curve analysis of *S. aureus* COL (black) and COL Δ*seb* (red), grown in triplicate; (**B**) TCA-precipitated supernatants (5 OD units) showing the exoprotein and superantigen profiles of *S. aureus* COL and COL Δ*seb* with detection of SEB production by anti-SEB antibodies using Western blot; (**C**) IL-2 production from DR4-B6 splenocytes activated with increasing concentrations of recombinant SEB, and bacterial supernatants diluted 1:10 from *S. aureus* COL and COL Δ*seb*. Results shown as the mean ± SEM from a representative data set.

To assess the role of SAgs in experimental *S. aureus* nasal colonization, a mutant strain of *S. aureus* COL with a deletion of the *seb* gene was generated as described in Materials and Methods. Growth curve analysis of the SEB deletion strain compared to the wild-type counterpart showed no obvious growth defects *in vitro* (Figure 1A). As expected, *S. aureus* COL Δ*seb* did not produce SEB as shown by the exoprotein profiles and Western blot analysis (Figure 1B). Additionally, we did not detect significant levels of IL-2 from DR4-B6 splenocytes treated with cultural supernatants from COL Δ*seb* compared to wild-type COL (Figure 1C). Although *S. aureus* COL also encodes *sei*, *sel-k* and *sel-x*, these data indicate that SEB is the dominant SAg produced by *S. aureus* COL *in vitro*. The *sea* deletion mutant was generated in *S. aureus* Newman as previously described, and similarly, has been characterized as lacking superantigenic activity [26].

### 2.2. Lack of SEA Transiently Increases S. aureus Newman Δsea Nasal Colonization

**Figure 2:**
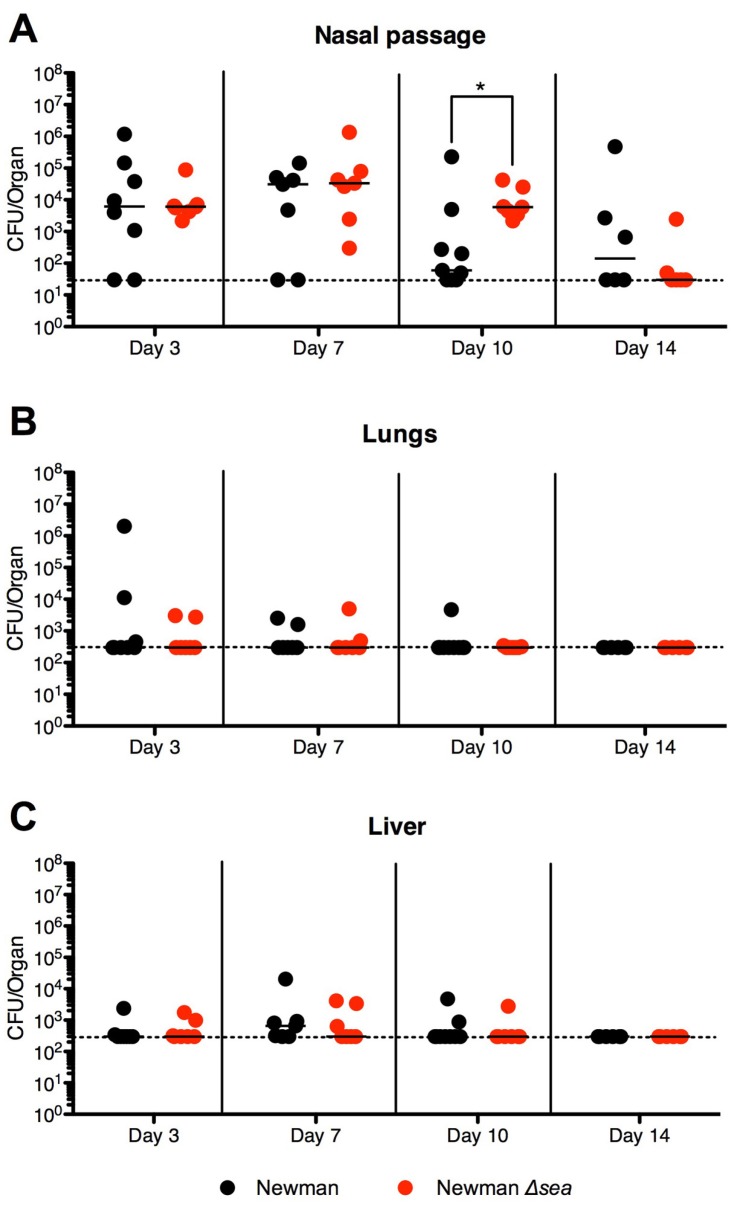
Nasal colonization of DR4-B6 mice with *S. aureus* Newman Δ*sea* results in a transient increase in bacterial load compared to wild-type Newman. DR4-B6 mice were infected nasally with 1 × 10^8^ CFUs of *S. aureus* Newman or Newman Δ*sea* (*n* = 6–9). Mice were sacrificed on days 3, 7, 10 and 14 and the (**A**) nasal passage; (**B**) lungs and (**C**) livers were assessed for overall *S. aureus* burdens. Each point represents an individual mouse and the line in each treatment group represents the median. The horizontal dotted line indicates the limit of detection. Data are pooled from of at least three independent experiments. Significant differences were determined by Mann-Whitney U test (*, *p* < 0.05).

To investigate if SEA plays a role during murine nasal colonization, DR4-B6 mice pre-treated with streptomycin (Sm) were inoculated with 1 × 10^8^ CFUs of *S. aureus* Newman or *S. aureus* Newman Δ*sea*. *S. aureus* was detected in the nasal passages of both *S. aureus* Newman- and *S. aureus* Newman Δ*sea*-infected mice up to day 14 post-inoculation. Generally, CFU counts were higher during the first week of colonization compared to the second week (Figure 2A). Infected mice did not show overt signs of infection (lack of piloerection, conjunctivitis, skin rashes, and dehydration, with normal activity levels), had no weight loss, and were generally healthy for the duration of the experiment (data not shown). Despite the apparent lack of infection, the lungs and livers of both infection groups revealed spread of bacteria beyond the nose, although the bacterial burdens in these organs were lower than in the nasal passage and generally very low by day 14 (Figure 2B,C). Bacteria were not detected in the kidneys or spleen (data not shown). No significant differences in bacterial loads were observed between bacterial strains on days 3 or 7 in the nasal passage. However, by day 10, *S. aureus* Newman Δ*sea*-colonized mice had increased CFUs compared to wild-type Newman-colonized mice (Figure 2A); however, this phenotype reverted to no differences between treatment groups by day 14. These data suggest that SEA does not play a major role during the initial stages of colonization, but may prevent higher bacterial densities from forming in the nose. While the lack of SEA production did allow higher bacterial densities to form, this transient difference did not result in better colonization at later time points, suggesting that it does not enhance the overall colonization capabilities of *S. aureus* Newman. No significant differences were observed in the spread of infection to other organs between wild-type and *sea*-null infections indicating that SEA likely does not influence dissemination in this model.

### 2.3. SEA Does Not Skew Vβ3 Subsets in vivo

**Figure 3:**
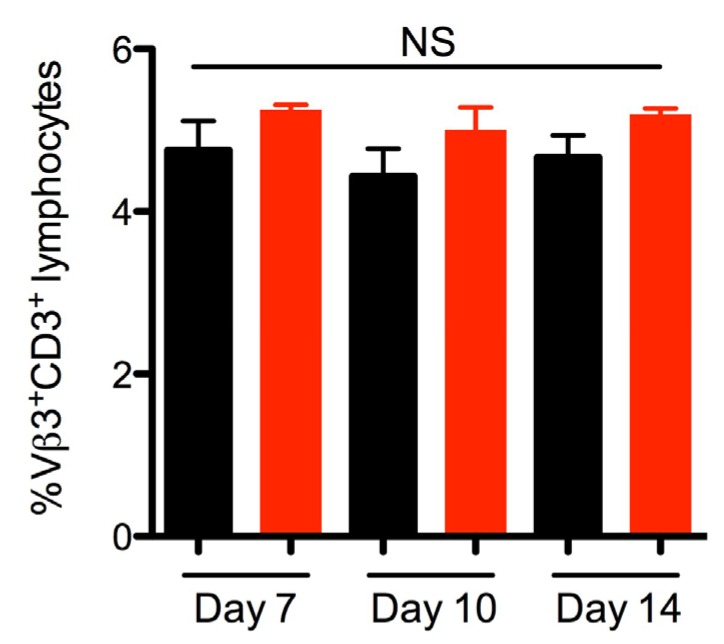
*S. aureus* Newman nasal colonization does not result in significant changes in the percentage of Vβ3^+^CD3^+^ T cells. Analysis of lymphocytes from lymph nodes isolated from DR4-B6 mice nasally inoculated with 1 × 10^8^ CFU *S. aureus* Newman or Newman Δ*sea* (*n* = 3–4). Cells were stained with antibodies against CD3 and Vβ3 and gated on CD3^+^ lymphocytes, followed by gating on the Vβ3^+^CD3^+^ population. Data are shown as the mean ± SEM and significant differences (*p* < 0.05) were determined by unpaired student’s *t*-test (NS = not statistically different).

We next aimed to evaluate if SEA was produced during *S. aureus* colonization by examining the Vβ profiles of infected mice. As SEA has been previously shown to skew murine Vβ3^+^ T cells during bacteremia [26], we analyzed the Vβ3 subset as well as levels of serum IgG against SEA in order to assess if SEA had *in vivo* activity. Analysis of the Vβ3^+^CD3^+^ lymphocytes from lymph nodes revealed no significant changes in this subset between *S. aureus* Newman or Newman Δ*sea*-inoculated mice on any of the days analyzed (Figure 3), although there was a slight trend of decreased Vβ3^+^ T cells in wild-type Newman-colonized mice. These data suggest that SEA may not be produced in large amounts, or is weakly active, during the length of the experiment. Additionally, no IgG against SEA could be detected in Newman and Newman Δ*sea*-inoculated mice sera (data not shown). Collectively, these data suggest that SEA was not produced in functional quantities *in vivo* during colonization. This may explain that lack of differences seen in bacterial burdens at earlier time points (Figure 2A), since the lack of SEA production by *S. aureus* Newman is functionally equivalent to infection with Newman Δ*sea*.

### 2.4. SEB Decreases Nasal Colonization

**Figure 4:**
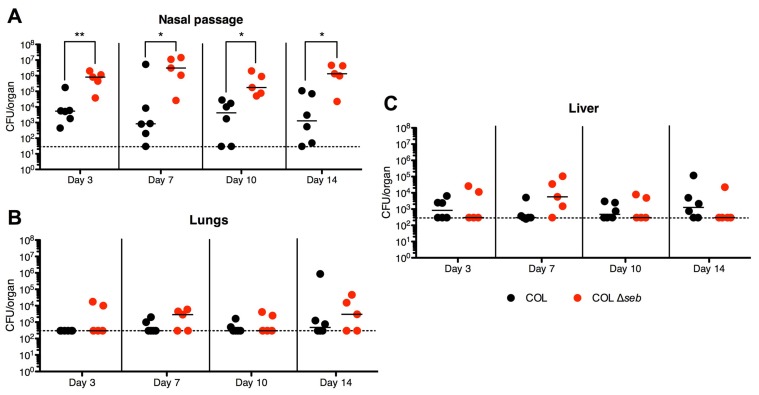
Murine nasal colonization with *S. aureus* COL Δ*seb* results in enhanced bacterial counts compared to wild-type COL. DR4-B6 mice infected nasally with 1 × 10^8^ CFUs of *S. aureus* COL (*n* = 6) or COL Δ*seb* (*n* = 5) were sacrificed on days 3, 7, 10 and 14. The (**A**) nasal passage, (**B**) lungs, and (**C**) livers were assessed for overall *S. aureus* loads. Each point represents an individual mouse and the line in each treatment group represents the median. The horizontal dotted line indicates the limit of detection. Data are pooled from of at least three independent experiments. Significant differences were determined by Mann-Whitney U test (*, *p* < 0.05; **, *p* < 0.01).

Unlike SEA, SEB is transcriptionally activated by the accessory gene regulator (*agr*) quorum-sensing system during exponential and late stages of growth [27] and this may also result in differential expression in response to environmental cues. Similar to colonization with *S. aureus* Newman, COL was found to colonize the nasal passages of infected mice in both treatment groups; however, colonization with wild-type *S. aureus* COL persisted with higher bacterial numbers (~10^3^–10^4^) (Figure 4A) compared to wild-type Newman (10^2^–10^3^) (Figure 2A), especially at later time points, suggesting that COL may be a better nasal colonizer than Newman in DR4-B6 mice. When mice were colonized with *S. aureus* COL Δ*seb*, bacteria recovered from the nasal passages were ~100-fold higher in CFUs at all time points compared with wild-type COL colonization alone (Figure 4A). As with nasal colonization by *S. aureus* Newman, all mice were apparently healthy for the duration of the experiment with no overt signs of infection. Spread of the infection to the lungs and livers was also observed during *S. aureus* COL and COL Δ*seb* colonization, although no significant differences were observed between CFUs of the two strains (Figure 4B,C). While a complete SAg-negative strain was not assessed (*i.e.*, *sel-k*, *sel-i* and *sel-x* are still encoded within COL), these data suggest that production of SEB actually inhibited high-density colonization within the nasal passage.

### 2.5. SEB Induces Late Vβ8 Skewing but not Anti-SEB IgG during Nasal Colonization

**Figure 5:**
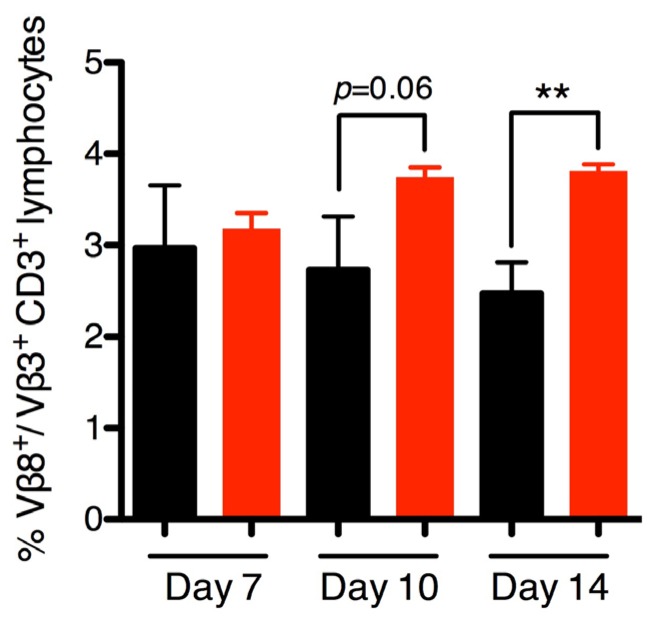
Staphylococcal enterotoxin B (SEB) is produced during *S. aureus* COL nasal colonization and specifically interacts with Vβ8^+^CD3^+^ lymphocytes. Lymphocytes from lymph nodes isolated from DR4-B6 mice nasally inoculated with 1 × 10^8^ CFU *S. aureus* COL or COL Δ*seb* were analyzed using flow cytometry (*n* = 3–5). Samples were stained with antibodies against either CD3 and Vβ3 or CD3 and Vβ8. Each mouse sample was stained with both Vβ3 and Vβ8, using Vβ3 as the internal control. Samples were gated on CD3^+^ lymphocytes, followed by gating on the Vβ3^+^CD3^+^ or Vβ8^+^CD3^+^ population and expressed as a ratio of Vβ8^+^ CD3^+^ to Vβ3^+^CD3^+^ cells per mouse. Data are shown as the mean ± SEM and significant differences determined by unpaired student’s *t*-test (**, *p* < 0.01).

To evaluate if the phenotype observed during *S. aureus* COL colonization was SEB-dependent, we assessed Vβ-skewing in mice colonized with wild-type *S. aureus* COL and COL Δ*seb* to test for functional SEB activity *in vivo*. It is well-established that SEB targets Vβ8.1/8.2^+^ (henceforth Vβ8^+^) T cells in mice [28] and Vβ3 was used as an internal control. The murine Vβ subsets targeted by SEl-K, SEl-I, and SEl-X are unknown to date and thus could not be assessed for *in vivo* activity although these strains showed no superantigenic activity *in vitro* (Figure 1C). While no differences could be detected early, by day 10 there was a trend of decreased Vβ8^+^ T cells, which was significantly decreased by day 14 (Figure 5). Interestingly, anti-SEB IgG antibodies were not detected from either COL- or COL Δ*seb*-colonized mice by day 14 (data not shown). The demonstrated Vβ-skewing by day 14 indicates that SEB was produced and functional during *S. aureus* COL nasal colonization. Furthermore, the difference in bacterial loads between COL and COL Δ*seb* (Figure 4A) at the early time points suggests that SEB was functioning early on during colonization, although we were not able to detect functional activity until the later time points.

## 3. Discussion

This is the first study where the role of SAgs has been directly and experimentally assessed during a controlled model of nasal colonization using SAg-sensitive, humanized transgenic mice. Our findings reveal that different SAgs may play distinctive roles during colonization as SEA only transiently altered CFUs for *S. aureus* Newman nasal colonization, while SEB production reduced *S. aureus* COL colonization throughout all experimental time points. Although *S. aureus* Newman also encodes *sel-x* and COL additionally encodes *sei*, *sel-k* and *sel-x*, the *in vitro* stimulation data suggest that in our growth conditions, these SAgs are not made in high quantities by these strains and thus may not play a role in our model. However, future studies should assess a complete SAg deletion strain in comparison to wild-type colonization.

Data from previous human studies suggest that SAgs may be involved during *S. aureus* colonization from two lines of evidence: real-time PCR analysis of nasal swabs from persistent carriers have demonstrated *in vivo* transcription of *sea* [23], and the finding that persistently-colonized individuals have increased levels of neutralizing antibodies against SEA and TSST-1 [20]. Although it has been suggested that non-*agr* regulated SAgs such as SEA may be involved during the early phases of colonization [29], this was not supported by our experimental model when we inoculated DR4-B6 mice with *S. aureus* Newman. SEA expression during Newman colonization is supported by the increase in bacterial colonization at day 10 by *S. aureus* Newman Δ*sea* despite the lack of significant Vβ-skewing. These data suggest that SEA was expressed in low amounts that transiently inhibited the formation of higher bacterial densities in the nasal cavities. Conversely, the decrease in Vβ8^+^ T cells during colonization with *S. aureus* COL compared to COL Δ*seb* mice is indicative of SEB expression by COL, which is responsible for the difference in nasal bacterial burdens. Direct comparison of the role of SEA *versus* SEB is difficult because they are encoded by two distinct strains. However, a notable difference between SEA and SEB lies in their regulation and expression: SEA is generally not produced in large amounts, whereas SEB production can reach high concentrations *in vitro* (Figure 1B), likely due to the activation of the *agr* two-component system. Thus, the high expression of SEB by *S. aureus* COL may have resulted in colonization with lower bacterial counts due to its inflammatory properties at all time points, while lower expression of SEA by *S. aureus* Newman did not have as dramatic differences. Although we have not genetically complemented the *S. aureus* COL Δ*seb* strain, compared with wild-type COL, other than production of SEB the exoprotein profiles are virtually indistinguishable.

The absence of anti-SAg IgG antibodies by day 14 is suggestive that either the SAgs were not processed as conventional antigens and presented to B cells, or that anti-SAg antibodies were not IgG isotypes and thus could not be detected by the assay employed. Human studies have concluded that colonization by *S. aureus* does not appear to induce a strong humoral response [23,30]. Thus, the high levels of anti-SEA antibodies in healthy subjects [20] may not be a result of persistent colonization, but rather breaches of the nasal mucosa from colonizing *S. aureus*, or skin infections. It has also been noted that anti-SAg antibodies are not always produced when the immune system is subjected to wild-type SAg, whereas SAg toxoids are much more immunogenic and are capable of forming robust anti-SAg antibodies [24,31], suggesting that SAgs can dysregulate the antibody response. Furthermore, it has been shown that naïve T cells exposed to SAgs will restrict antibody production, but will not affect ‘primed’ T cells [32], which may explain the lack of anti-SAg IgG in our colonized mice. TSS patients that fail to seroconvert after an episode may lead to recurrence, which has been attributed to the mechanisms of TSST-1 that prevent the development of Th2 responses, and thus T-cell dependent B cell activation [33,34].

Our study was extended to 14 days to observe differences in dissemination to other organs. *agr*-regulated SAgs such as SEB and TSST-1 may be involved in dissemination from the main bacterial colony, during which many exoproteins and virulence factors are produced, as opposed to cell-surface factors such as MSCRAMMs that are primarily involved in the initial colonization phase [29]. Surprisingly, we found bacteria in the lungs and livers of colonized mice as early as after three days, even though the mice did not show any overt signs of infection. However, there were no significant differences in the bacterial loads in these extra-nasal locations between the wild-type strains and their SAg deletion counterparts, suggesting that neither SEA nor SEB were involved in dissemination from the nasal cavity.

While SAgs are generally thought to enhance virulence [16,35], including the development of toxic shock syndrome [36], the deletion of SAgs actually increased staphylococcal CFUs in the nasal cavity. Interestingly, although colonization with *S. aureus* Newman Δ*sea* resulted in higher bacterial counts at day 10, this did not translate into long-term fitness and actually decreased back to wild-type levels by day 14. This suggests that higher bacterial densities in the nose may not be beneficial for asymptomatic colonization. Extending the length of the study may further clarify this theory since COL Δ*seb* maintained a higher bacterial density throughout the duration of the experiment. Although we did not observe differences in dissemination in our model during *S. aureus* COL and COL Δ*seb* colonization, the highest bacterial counts in the lungs were mostly COL Δ*seb*-inoculated mice, suggesting increased seeding from the higher bacterial counts in the nasal cavity. Given that bacteria colonizing the anterior nares are poised for both transmission between people and dissemination within the host, the vestibulum nasi is a desirable environment for *S. aureus* to reside in. Thus, *S. aureus* may utilize SAgs to prevent nasal bacteria from overwhelming this niche and breaching the mucosa, potentially leading to elimination by the immune system, thus acting as ‘checkpoints’ of dissemination. Since higher densities of bacteria may result in a greater inflammatory response, maintaining a low presence in the nose may be an evolutionarily prudent tactic to maintain long-term asymptomatic colonization. This is supported by the low bacterial burdens isolated from human nasal carriers during asymptomatic colonization [25]. Thus, this work supports the clinical finding that SAgs are expressed during nasal colonization [23], and in the context of colonization, these toxins may play an important role for influencing bacterial densities during this commensal lifestyle. This provides evidence for a novel role for SAgs, contrary to the traditional role of having been associated with enhancing virulence in severe invasive diseases.

## 4. Experimental Section

### 4.1. Mice

Six-to-twelve week old male and female HLA-DR4-IE (DRB1 * 0401) humanized transgenic mice lacking endogenous mouse MHC-II on a C57BL/6 (B6) background [37] (herein referred to as DR4-B6 mice) were used for all *in vivo* infection experiments. B6 mice were purchased from Charles River. All animal experiments were performed according to protocols approved by the Animal Use Subcommittee at Western University and in accordance with the Canadian Council on Animal Care Guide to the Care and Use of Experimental Animals.

### 4.2. Bacterial Strains, Media and Growth Conditions

*Escherichia coli* DH5α was used as a cloning host, grown in Luria Bertani (LB) broth (Difco; Mississauga, ON, Canada) at 37 °C with shaking at 250 rpm and supplemented with 150 μg/mL ampicillin when necessary. Strains of *S. aureus* listed in Table 1 were grown in tryptic soy broth (TSB) (Difco) at either 30 °C or 37 °C with shaking, and supplemented with appropriate antibiotics (Sigma Aldrich; Oakville, ON, Canada). Growth curves were performed using a Bioscreen C MBR system (Thermo Labsystems; Milford, MA, USA).

**Table 1:** Bacterial strains used in this study.

Strain	Description	Source
*S. aureus* Newman	Early methicillin sensitive isolate from secondary infection in a patient with tubercular osteomyelitis (Sm sensitive)	[38]
*S. aureus* Newman SmR	*S. aureus* Newman resistant to Sm	This study
*S. aureus* Newman SmR Δ*sea*	*sea*-null *S. aureus* Newman (with resistance to Sm)	[26]
*S. aureus* RN4220	Restriction-deficient derivation of NCTC8325-4	[39]
*S. aureus* COL	Early methicillin-resistant strain of *S. aureus* isolated in the 1960s	[40]
*S. aureus* COL Δ*seb*	*seb* deletion strain of *S. aureus* COL	This study
*E. coli* DH5α	Cloning strain	Invitrogen

### 4.3. Selection of a Streptomycin-Resistant S. aureus Strain

*S. aureus* strain Newman is a methicillin-sensitive clinical isolate from the 1950s that is commonly used in experimental studies of staphylococcal pathogenesis [38]. Initial attempts to colonize mice resulted in competition with endogenous bacterial species and poor *S. aureus* colonization. This phenomenon has been documented previously in the literature [41] and represents an additional challenge for *S. aureus* to colonize in nature. However, for the purposes of testing our hypothesis, an antibiotic dosing regime was instated with streptomycin sulfate (Sm) in order to reduce the endogenous murine microbiota, as previously described [41]. Since *S. aureus* Newman is not naturally resistant to Sm, a Sm-resistant strain was generated by plating *S. aureus* Newman on Sm gradient TSA plates and selecting for bacteria with increased resistance. *S. aureus* Newman SmR was able to be grown in TSB containing 500 μg/mL Sm. No loss of resistance was observed after daily 1% subcultures in TSB without Sm for up to 6 days. Since the growth rate was reduced with the inclusion of Sm, preparations of bacteria for inoculation into mice were cultured without Sm. *spa* genotyping [42] showed that *S. aureus* Newman SmR had the same genetic background as Sm-sensitive Newman and qRT-PCR showed normal levels of *sea* expression (data not shown). The *sea* gene was deleted in the Newman SmR background to maintain isogenicity as described [26]. For the remainder of the experiments, Newman SmR will be referred to as Newman and the isogenic *sea* deletion strain as Newman Δ*sea*.

### 4.4. Construction of S. aureus COL *Δ*seb

*S. aureus* COL is one of the earliest MRSA strains to be isolated in the 1960’s and data mining of the sequenced COL genome [40] revealed the SAgs: SEB, SEl-K and SEl-I (formerly SEQ [43]) as well as SEl-X. COL was found to be inherently resistant to Sm and thus did not require a new Sm-resistant strain to be generated. A markerless deletion was created in *seb* based on previously described methods [44]. Briefly, a 524-bp fragment upstream of *seb* was amplified using the primers 5'-TAGGGATCCAGCTCGTGATATGTTGGGTAAA-3' and 5'-GGGCGGGTCGACTGAAATAAA TAATCTCTTATACA-3' along with a 505 bp region downstream of *seb* amplified by the primers 5'-CGATGTCGACTATCTTACGACAAAGAAAAAGTGAAA-3' and 5'-TCAGGAATTCGAGATGC TTTGAAAGAAGCAAA-3'. These products were directionally cloned into pMAD, creating pMAD::*seb* which only includes 54 bp of the original 801 bp encoding *seb*. This knockout construct was methylated by *S. aureus* RN4220 and electroporated into *S. aureus* COL. To create the *seb* knockout, a single-integration event was first isolated, followed by subcultures in TSB without antibiotics grown at 30 °C. Since pMAD contains β-galactosidase, patching of white colonies detected colonies that had lost resistance to erythromycin, evident of plasmid curing and screened by PCR to verify successful deletion of *seb*.

### 4.5. Detection of SAgs in Cultural Supernatants in vitro

Bacterial cultures were grown overnight in TSB, cells were pelleted, and cell-free supernatants equivalent to 5.0 OD_600_ units of culture were collected. Proteins were precipitated with 10% trichloroacetic acid (TCA) overnight on ice, washed twice with ice-cold 70% ethanol and resuspended in Laemmli buffer as previously described [45]. Samples were analyzed on 12% polyacrylamide gels stained with Coommassie Brilliant Blue R-250. For Western blot analysis of SEB expression, samples were transferred to polyvinylidene difluoride (PVDF) membranes (Millipore; Etobicoke, ON, Canada) at 100V for 1 h. The membrane was blocked at roomed temperature for 1 h with PBS supplemented with 10% skim milk and 5% horse serum (Gibco; Burlington, ON, Canada). Following removal of the blocking buffer, the membrane was incubated with rabbit polyclonal anti-SEB antibodies (kindly provided by Patrick Schlievert, University of Iowa, IA, USA) diluted 1:100 in PBS supplemented with 5% skim milk and 2.5% horse serum. The membrane was washed three times with PBS supplemented with 0.02% Tween-20 (Fisher Scientific; Ottawa, ON, Canada) (PBST), followed by incubation with IRDye-conjugated goat anti-rabbit secondary antibody (LI-COR Biosciences; Lincoln, NB, USA) diluted 1:10,000 in PBST supplemented with 5% skim milk and 2.5% horse serum for 1 h in the dark. The membrane was imaged using an Odyssey imager (LI-COR Biosciences).

### 4.6. Assessment of Superantigenic Activity of S. aureus COL Strains in vitro

Supernatants from *S. aureus* strains were tested for SAg activity using DR4-B6 splenocytes seeded into 96-well plates as described above. Titrations of recombinant SEB, and supernatants from overnight cultures of *S. aureus* COL and COL Δ*seb* were diluted 1:10 were added to splenocytes for 18 h at 37 °C, and supernatants were assayed for IL-2 by ELISA according to manufacturer’s instructions (eBioscience; San Diego, CA, USA).

### 4.7. Staphylococcus aureus Nasal Colonization Model

Twenty-four hours prior to inoculation, mice were administered drinking water supplemented with 2.0 mg/mL of Sm *ad libitum*, which was changed every 3–4 days for the duration of the experiment. Bacteria picked from a TSA plate were grown in 5 mL TSB overnight (16–18 h), OD_600_ was adjusted to 1.0, subcultured 2% into 50 mL TSB and grown to OD_600_ ~ 3.0–3.5. The bacterial pellet was washed 3 times with Hank’s Buffered Salt Solution (HBSS) (Hyclone; Logan, UT, USA) and suspended at a concentration of 1 × 10^10^ CFU/mL in HBSS. Isofluorane-anesthetized mice were nasally inoculated by slowly pipetting 5 μL into each nare and allowing the animal to breathe in the suspension naturally, resulting in a total inoculum of 1 × 10^8^ CFU *S. aureus* per mouse. Mice were weighed and monitored daily according to animal ethics use protocol and sacrificed at days 3, 7, 10, and 14. To enumerate the amount of bacteria in the nose, euthanized mice were decapitated and the lower jaws removed. The entire snout was excised using the back of the mouth opening as an anatomical marker in order to include any bacteria in the nasal passage. The whiskers and surrounding skin were removed without touching the nose, and the remaining tissue was collected in HBSS. The kidneys, hearts, lungs, livers and spleens were also collected and all organs were homogenized and serially diluted and plated on MSA (Difco) to differentiate between *S. aureus* and any endogenous bacteria. *S. aureus* CFUs were not different between plates containing Sm and without Sm (data not shown), thus Sm was not included in plates. Plates were enumerated after being incubated at 37 °C for 24 h. Counts less than 3 CFU/100 µL were considered below the detectable limit.

### 4.8. Determination of SAg Function in vivo

Lymph nodes (cervical, axillary, brachial, inguinal, and popliteal) were isolated *in toto* from mice and pushed through a cell strainer to create a single cell suspension in PBS. Cells were stained with APC-conjugated anti-CD3 (clone 145-2C11) (eBioscience) and FITC-conjugated anti-Vβ3 (clone KJ25) (BD Pharmingen; Mississauga, ON, Canada) or FITC-conjugated anti-Vβ8 (clone KJ16) (eBioscience) and assayed by flow cytometry. Data were analyzed using FlowJo v.8.7. (Treestar; Ashland, OR, USA).

### 4.9. Statistical Analyses

All statistical analyses were performed using Prism v5.0 (GraphPad; La Jolla, CA, USA) with *p* < 0.05 being considered significant.
